# Physical Exercise: A Promising Treatment Against Organ Fibrosis

**DOI:** 10.3390/ijms26010343

**Published:** 2025-01-02

**Authors:** Xiaojie Ma, Bing Liu, Ziming Jiang, Zhijian Rao, Lifang Zheng

**Affiliations:** 1 College of Physical Education, Shanghai University, Shanghai 200444, China; maxiaojie@shu.edu.cn (X.M.); lb1319@shu.edu.cn (B.L.); shujzm@shu.edu.cn (Z.J.); 2 College of Physical Education, Shanghai Normal University, Shanghai 200234, China; 3 Exercise Biological Center, China Institute of Sport Science, Beijing 100061, China

**Keywords:** fibrosis, exercise, therapeutics, mechanism

## Abstract

Fibrosis represents a terminal pathological manifestation encountered in numerous chronic diseases. The process involves the persistent infiltration of inflammatory cells, the transdifferentiation of fibroblasts into myofibroblasts, and the excessive deposition of extracellular matrix (ECM) within damaged tissues, all of which are characteristic features of organ fibrosis. Extensive documentation exists on fibrosis occurrence in vital organs such as the liver, heart, lungs, kidneys, and skeletal muscles, elucidating its underlying pathological mechanisms. Regular exercise is known to confer health benefits through its anti-inflammatory, antioxidant, and anti-aging effects. Notably, exercise exerts anti-fibrotic effects by modulating multiple pathways, including transforming growth factor-β1/small mother decapentaplegic protein (TGF-β1/Samd), Wnt/β-catenin, nuclear factor kappa-B (NF-kB), reactive oxygen species (ROS), microRNAs (miR-126, miR-29a, miR-101a), and exerkine (FGF21, irisin, FSTL1, and CHI3L1). Therefore, this paper aims to review the specific role and molecular mechanisms of exercise as a potential intervention to ameliorate organ fibrosis.

## 1. Introduction

Fibrosis represents a prevalent feature across a spectrum of chronic diseases. With 45% of deaths in developed countries closely linked to the development of fibrosis, the treatment of organ fibrosis is one of the critical challenges facing global public health. Recognizing the complex nature of treating organ fibrosis, urgent attention is warranted [1,2]. The pivotal role of exercise in ameliorating various chronic diseases has garnered widespread recognition [3]. Research indicates its capacity to enhance prognosis and quality of life in patients across 26 chronic diseases [4]. Notably, chronic diseases, including chronic kidney disease (CKD) [5], lung disease (silicosis) [6], liver disease (NAFLD, NASH) [7,8], and skeletal muscle disease (sarcopenia [9,10], muscle damage [11]), exhibit a fibrotic phenotype, which exercise may potentially mitigate. While fibrogenesis mechanisms vary by organ, inflammation, oxidative stress, and aging emerge as common denominators in fibrosis pathogenesis [12]. Extensive studies underscore exercise’s multifaceted benefits, including potent anti-inflammatory [13,14,15,16], antioxidant [13,17,18], anti-aging [19,20], metabolism-regulating [21,22,23], anti-apoptotic [13,24,25], and immuno-modulatory [26,27] properties. However, it is important to note that much of the current evidence, particularly in relation to molecular mechanisms and signaling pathways, stems from animal models, primarily in mice and rats. While these models provide valuable insights, their translational relevance to human fibrosis warrants careful interpretation. A wealth of literature supports exercise’s efficacy in mitigating fibrosis across organs such as the heart, lungs, kidneys, liver, and skeletal muscle by modulating diverse signaling pathways [15,19,28,29,30]. This collective evidence suggests exercise as a promising and effective anti-fibrotic intervention. Therefore, this paper aims to review the role of exercise and its potential molecular mechanisms in ameliorating fibrosis across multiple organs, with particular attention on findings derived from animal studies.

## 2. The Pathogenesis of Fibrosis

**Figure 1:**
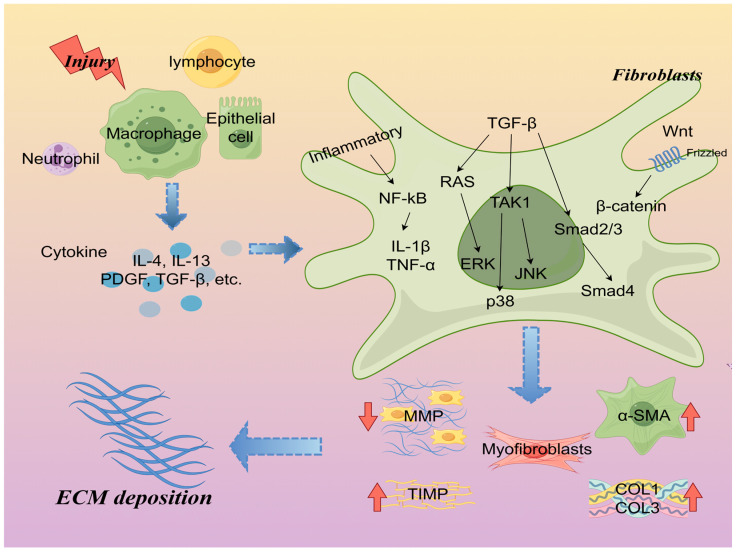
The pathogenesis of fibrosis: After tissue injury, immune cells (mainly macrophages) are activated and release cytokines (e.g., IL-4, IL-13, PDGF, TGF-β, etc.). Through signaling pathways such as TGF-β/Smad and Wnt/β-catenin, fibroblasts are transformed into myofibroblasts, and myofibroblasts produce a large amount of ECM, leading to the generation of fibrosis. IL-4: interleukin-4; IL-13: interleukin-13; IL-1β: interleukin-1β; TGF-β: transforming growth factor-β; smad: small mother decapentaplegic protein; PDGF: platelet-derived growth factor-D; ERK: extracellular signal-regulated kinase; p38: p38 mitogen-activated protein kinases; JNK: c-Jun N-terminal kinases; TAK1: transforming growth factor-β (TGF-β)-activated kinase 1; α-SMA: α-smooth muscle actin; COL1: Collagen 1; COL3: Collagen 3; MMP: matrix metallopeptidase; TIMP-1: tissue inhibitor of metal protease1. Created with Figdraw (www.figdraw.com), license ID: TTWOW4366f.

Fibrosis is a chronic progressive disease characterized by an excessive deposition of extracellular matrix (ECM) and scar tissue formation. Organ fibrosis is the process by which normal tissue cells in an organ are replaced by fibrous tissue, and it is also the result of dysfunctional wound healing after tissue damage, typically associated with chronic inflammation and tissue damage resulting from specific pathological conditions, such as chronic infections, autoimmune disorders, or metabolic syndromes. Fibrosis can impair normal physiological functions of the organ and is associated with a poor prognosis for the patient [31]. Studies have evidenced fibrosis occurrence in various organs, such as the heart, kidneys, lungs, liver, and skeletal muscle [32]. Meanwhile, many cellular, human, and animal models have been developed over the past five decades. As in other areas of biomedical research, mice/rats have served as primary research subjects due to their rapid modeling, ability to be genetically manipulated, and low cost [33]. The general mechanism of fibrosis pathogenesis involves a plethora of chemical signals and cytokines. Upon tissue damage, immune cells, predominantly macrophages, become activated and release numerous mediators, including interleukin-4, interleukin-13, interleukin-25, interleukin-33, platelet-derived growth factor (PDGF), cytokines, and chemokines, which activate fibroblasts and promote their transformation into myofibroblasts. Myofibroblasts, characterized by surface markers such as α-smooth muscle actin (α-SMA), contribute to ECM production and accelerate fibrosis by inducing contractility and activating TGF-β1 [34]. In this process, the degradation of the ECM is of particular importance. Extracellularly, the balance between matrix metalloproteinases (MMPs) and tissue inhibitors of metalloproteinases (TIMPs) is a key pathway for accelerated ECM degradation, which, in addition to the extracellular pathway, can be carried out by macrophages and myofibroblasts through the internalization of intact or fragmented collagen [35]. Moreover, a myriad of factors, including cytokines (TNF-α, IL-1β, IL-6), chemokines (MCP-1, CXCL5), growth factors (PDGF, CTGF, TGF-β1), and reactive oxygen species (ROS), co-regulate the process of organ or tissue fibrosis [2,12]. Notably, TGF-β1, a prototypical pro-fibrotic factor, orchestrates fibrosis via canonical Smad-dependent [36,37] and non-Smad-dependent pathways involving extracellular signal-regulated kinase (ERK), p38 mitogen-activated protein kinase (p38MAPK), and others [38]. Additionally, the Wnt/β-catenin signaling pathway assumes significance in fibrosis regulation [39] (Figure 1).

## 3. Exercise Regulates Organ Fibrosis

### 3.1. Pulmonary Fibrosis

#### 3.1.1. Effects of Exercise on Pulmonary Fibrosis

Pulmonary fibrosis is a chronic, progressive respiratory disease [40] that culminates in extensive lung tissue damage, respiratory dysfunction, and the eventual loss of lung function [41]. While treatments such as pirfenidone, nintedanib, and lung transplantation are commonly utilized [42], their high costs impose significant financial burdens. Exercise, a non-pharmacological intervention for health promotion, has shown promise in improving lung function and cardiorespiratory endurance in patients with pulmonary fibrosis [43]. Animal studies have demonstrated that aerobic exercise significantly attenuates lung inflammation, oxidative stress, apoptosis, and fibrosis induced by chronic obstructive pulmonary disease (COPD) [13]. Notably, swimming exercise has been particularly effective in ameliorating bleomycin (BLM)-induced pulmonary fibrosis in mice [44,45]. Collectively, these studies underscore the effectiveness of exercise in delaying or improving pulmonary fibrosis.

#### 3.1.2. Mechanisms of Exercise Regulation of Pulmonary Fibrosis

Lung epithelial cell injury and dysfunction play pivotal roles in the development of lung fibrosis, accompanied by the activation of signaling pathways such as TGF-β1 and Wnt during the repair process [46]. Research has shown that aerobic exercise reduces collagen deposition and fibrosis by inhibiting the Wnt/β-catenin signaling pathway and attenuating alveolar epithelial–mesenchymal transition (EMT), inflammation, and oxidative stress [28]. Hydrogen sulfide (H2S) is an endogenous gas neurotransmitter with anti-inflammatory and antioxidant properties. It also protects against various respiratory diseases, such as chronic obstructive pulmonary disease (COPD) [47], asthma [48], and pulmonary fibrosis [49]. One study indicated that aerobic exercise (45 min/day, five times/week for 4 weeks) induced an upregulation of endogenous H_2_S [50] and decreased activity in the TGF-β1/Smad and low-density lipoprotein receptor-related protein (LRP-6)/β-catenin signaling pathways, inhibiting EMT and thereby attenuating BLM-induced lung fibrosis [50]. Moreover, idiopathic pulmonary fibrosis (IPF) is a chronic fibrosing interstitial pneumonia characterized by abnormal serotonin (5-hydroxytryptamine [5-HT]) and protein kinase B (Akt) signaling. Aerobic training (60 min/day, five times/week for 4 weeks) improves BLM-induced pulmonary fibrosis by inhibiting 5-HT and Akt levels [51]. However, El-Mafarjeh E et al. showed that four weeks of moderate-intensity aerobic exercise (60 min/day, five times/week for 4 weeks) only attenuated BLM-induced lung tissue inflammation in mice and did not improve pulmonary fibrosis [52]. Therefore, whether aerobic exercise has an ameliorative effect on BLM-induced pulmonary fibrosis needs to be further investigated.

Silicosis, also known as pneumoconiosis, is a systemic disease characterized by diffuse fibrosis of the lung tissue caused by the long-term inhalation of production dust. Silica induces silicosis and leads to abnormally elevated levels of interleukin-17A (IL-17A) in lung tissue. Studies have shown that 4 weeks of moderate-intensity aerobic exercise suppresses the expression of TGF-β1 and inflammatory pathways TLR4/TNF-α and SRB/NLPR3, which, in turn, suppresses the expression of macrophage-derived IL-17A, thereby ameliorating silica-induced pulmonary fibrosis [6]. In addition, IL-17A acts synergistically with TNF-α, with both inducing elevated levels of CXC motif chemokine ligand 5 (CXCL5) expression in alveolar epithelial cell Ⅱ [53]. Aerobic exercise for 4 weeks ameliorated silica-induced lung fibrosis in mice through the inhibition of the IL-17A/CXCL5/Chemokine (C-X-C motif) Receptor 2 (CXCR2) axis [6]. Thus, moderate-intensity aerobic exercise (60 min/day, five times/week for 4 weeks) attenuates silica-induced lung fibrosis by inhibiting the TLR4-TNF-α, SRB-NLPR3, TGF-β1, and IL-17A/CXCL5/CXCR2 signaling pathways.

**Figure 2:**
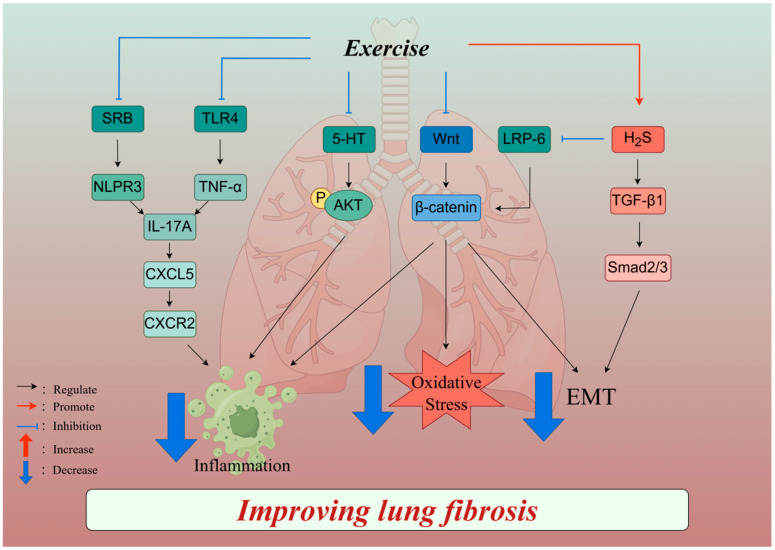
The mechanisms of exercise modulation of pulmonary fibrosis. Exercise ameliorates paraquat-induced pulmonary fibrosis by impeding the Wnt/β-catenin pathway, dampening inflammation, oxidative stress, and EMT. In bleomycin-induced pulmonary fibrosis, exercise alleviates fibrosis by enhancing endogenous hydrogen sulfide (H_2_S) synthesis, thereby inhibiting the LRP-6/β-catenin and TGF-β1 signaling pathways or reducing lung inflammation and EMT via the suppression of serotonin (5-HT) and Akt phosphorylation. Additionally, in silica-induced silicosis, exercise attenuates lung fibrosis by suppressing the TLR4-TNF-α and SRB-NLRP3 pathways and further inhibiting the IL-17A-CXCL5-CXCR2 inflammatory axis. SRB: scavenger receptor B; NLPR3: NOD-like receptor thermal protein domain associated protein 3; TLR4: Toll-like receptor 4; TNF-α: tumor necrosis factor; IL-17A: interleukin-17A; CXCL5: CXC motif chemokine ligand 5; CXCR2: Chemokine (C-X-C motif) Receptor 2; 5-HT: serotonin; AKT: protein kinase B; LRP-6: low-density lipoprotein receptor-related proteins; H_2_S: hydrogen sulfide; TGF-β1: transforming growth factor-β1; smad: small mother decapentaplegic protein. Created with Figdraw (www.figdraw.com), license ID: PYPIP737ba.

In summary, aerobic exercise mitigates pulmonary fibrosis caused by bleomycin and silica by suppressing inflammation, oxidative stress, and EMT. These findings emphasize the multifaceted benefits of aerobic exercise in combating pulmonary fibrosis (Figure 2). However, there is currently no literature reporting whether other forms of exercise, such as resistance training and high-intensity interval training, have a beneficial effect on bleomycin- and silica-induced pulmonary fibrosis; thus, further exploration is needed.

### 3.2. Renal Fibrosis

#### 3.2.1. Effect of Exercise on Renal Fibrosis

Renal fibrosis, a common pathological feature in the progression of various chronic kidney diseases to end-stage renal disease, such as hypertensive nephropathy and diabetic nephropathy, is characterized by excessive extracellular matrix deposition, a loss of renal parenchyma, and interstitial fibrosis [54], ultimately leading to renal dysfunction. Inflammation [55,56], oxidative stress [57,58,59], fibroblast transdifferentiation [60], and a reduction in peritubular capillaries are pivotal in renal fibrosis [61]. Research indicates that exercise can effectively improve blood pressure and renal function in chronic kidney disease patients [5,62]. Animal studies have further demonstrated that various exercise forms mitigate renal fibrosis by reducing the expression of inflammatory factors, collagen, and matrix metalloproteinases while enhancing the activity of antioxidant enzymes [63,64,65,66,67]. This underscores the role of exercise in attenuating renal fibrosis through its anti-inflammatory and antioxidant effects.

#### 3.2.2. Mechanisms of Exercise Regulation of Renal Fibrosis

Renal fibrosis and scar tissue formation are pivotal factors in the progression of diabetic nephropathy to end-stage renal disease. Sirtuins belong to an evolutionarily conserved family of NAD+-dependent deacetylases, and alterations in sirtuin expression are crucial in many diseases, including metabolic syndrome, diabetes, cancer, and aging. In the kidney, the most widely studied deacetylase is SIRT1, which exerts a protective effect by inhibiting cell apoptosis, inflammation, and fibrosis [68]. Current research has identified exercise as a cost-effective and low-risk approach to improve diabetic nephropathy [69,70]. Four weeks of aerobic exercise (45 min/day, five times/week for 4 weeks) ameliorates glomerular fibrosis in diabetic mice by enhancing endogenous H_2_S production in renal tissues and regulating the Sirt1/p53 signaling pathway to inhibit apoptosis [71]. Another study found that 6 weeks of aerobic exercise (60 min/day, six times/week for 7 weeks) improved mitochondrial dysfunction and reduced renal fibrosis in mice with type 1 diabetes by activating the Sirt1/PGC-1α signaling pathway [65]. A recent study demonstrated that 8 weeks of aerobic exercise (30–50 min/day, six times/week for 8 weeks) improved renal interstitial fibrosis in type 2 diabetes (T2DM) mice by inhibiting the TGF-β1/Smad3 pathway through the upregulation of Sirt1 expression [72]. This suggests that Sirt1 may be a key target for aerobic exercise to ameliorate diabetic renal fibrosis. Moreover, inflammation and oxidative stress are critical factors in the development of diabetic renal fibrosis. NADPH oxidase 4 (NOX4), an enzyme closely related to oxidative stress, is the primary source of ROS in the kidney, and upregulation of the NOX4 expression level can lead to the overproduction of ROS under high glucose conditions. Excess ROS induce the activation of the NLPR3 inflammasome through the activation of the NF-κB pathway to promote renal inflammation and fibrosis [73,74]. Research has demonstrated that 8 weeks of aerobic exercise (60 min/day, five times/week for 8 weeks) reduces renal oxidative stress and inflammation, ameliorating renal fibrosis in db/db mice by modulating the NOX4/ROS/NF-κB/NLPR3 pathway [18]. Furthermore, Boor et al. found that 10 weeks of moderate-intensity exercise significantly ameliorated advanced glycation end products (AGEs)-induced renal tubulointerstitial fibrosis in Zucker rats [75], accompanied by a decreased expression of TGF-β1, platelet-derived growth factor receptor-β (PDGFR-β), and COL-I in the renal tubular interstitium. However, our previous study found that 8 weeks of high-intensity interval training (60 min/day, five times/week for 8 weeks) exacerbated renal injury and fibrosis in T2DM mice by activating the phosphatidylinositol 3-kinase (PI3K)/AKT/mammalian target of rapamycin (mTOR) signaling pathway and upregulating the expression of fibrosis-related proteins (TGF-β1, CTGF, collagen-III, α-SMA) [76]. These findings suggest that moderate-intensity aerobic exercise attenuates diabetes-induced renal fibrosis by attenuating inflammation, oxidative stress, and the TGF-β1/Smad pathway, whereas high-intensity exercise exacerbates it.

Hypertension significantly contributes to cardiovascular and cerebrovascular pathogenesis and is a primary cause of chronic kidney injury. Hypertensive nephropathy is the second leading cause of end-stage renal disease after diabetes [77]. Hypertensive nephropathy is characterized by interstitial fibrosis and the infiltration of inflammatory cells, with extensive lesions in epithelial renal tubular cells [78]. Research has demonstrated that 8 weeks of swimming exercise (60 min/day, six times/week for 8 weeks) effectively ameliorates renal fibrosis in spontaneously hypertensive rats by inhibiting the TGF-β1/Smad pathway [79]. Moreover, elevated expression levels of inflammation-associated proteins like interleukin-6 (IL-6) and cyclooxygenase-2 (COX-2), along with fibrosis-associated proteins (TGF-β, p-Smad2/3, CTGF, MMP9, MMP2), were observed in the kidneys of spontaneously hypertensive rats. Twelve weeks of aerobic exercise (60 min/day, five times/week for 12 weeks) significantly mitigated the expression of these factors, thereby improving renal cortical inflammation and fibrosis in hypertensive rats [80]. Connective tissue growth factor (CTGF) plays a crucial role in the TGF-β/Smad3 signaling pathway, mediating EMT and accelerating angiotensin II (Ang II)-induced renal fibrosis [81,82,83]. One study indicated that 14 weeks of moderate-intensity aerobic exercise (60 min/day, five times/week for 14 weeks) inhibited CTGF and α-SMA expression, blocked the Ang II-angiotensin II type I receptor (AT1R)-TGF-β signaling pathway, and effectively ameliorated renal fibrosis in spontaneously hypertensive rats [84]. Furthermore, it also suggested that moderate-intensity exercise yielded superior results compared to low-intensity exercise in attenuating renal fibrosis [84]. Conversely, high-intensity exercise was found to exacerbate renal fibrosis in spontaneously hypertensive rats. Zhao et al. demonstrated that 14 weeks of high-intensity exercise (60 min/day, five times/week for 14 weeks) activated the transient receptor potential vanilloid 4 (TRPV4)-TGF-β1-Smad2/3-CTGF signaling pathway through elevated lactic acid levels, leading to worsened renal fibrosis [29]. These findings underscore the differential effects of exercise intensity on hypertensive nephropathy-induced renal fibrosis, with moderate-intensity aerobic exercise offering greater protection and high-intensity exercise exacerbating the condition.

In addition, aging-induced reduction in autophagic activity is closely related to the onset and progression of fibrosis [85]. Kim et al. [86] discovered that the TGF-β1/activated kinase 1 (TAK1)/mitogen-activated protein kinase (MAPK) kinase (MKK3)/p38MAPK signaling pathway is involved in the induction of autophagy. Incremental loading exercise (15–60 min/day, five times/week for 6 weeks) has been shown to ameliorate renal fibrosis in aged mice by reducing extracellular matrix accumulation and delaying epithelial–mesenchymal transition through the modulation of the TGF-β1/TAK1/MKK3/p38MAPK signaling pathway and enhanced autophagy activation [87]. Moreover, the underlying molecular mechanism of aging leading to renal fibrosis is also related to lipid accumulation [88]. A 12-week swimming exercise (60 min/day, six times/week for 12 weeks) improved renal fibrosis by inhibiting the expression of miR-21 and miR-34a, thus activating PPAR α to reduce oxidative stress, inflammation, and lipid accumulation in the kidneys of aged rats [89]. These studies indicate that exercise primarily improves age-related renal fibrosis by modulating inflammation, oxidative stress, autophagy activity, and the TGF-β1 pathway.

**Figure 3:**
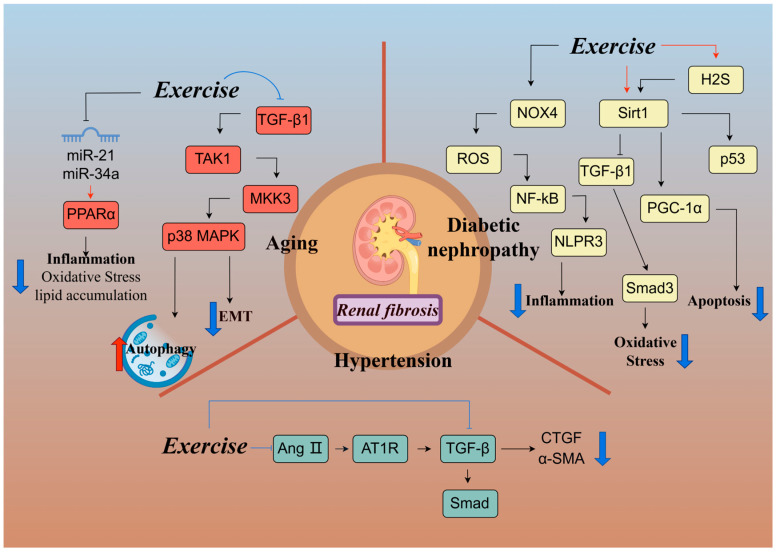
Mechanisms of exercise modulation of renal fibrosis. Exercise exerts its beneficial effects on renal fibrosis through various mechanisms: Exercise suppresses NOX4-dependent ROS production in the kidney, thereby inhibiting the NF-κB/NLPR3 inflammasome pathway, or by targeting Sirt1, which ultimately ameliorates renal fibrosis associated with diabetic nephropathy. Exercise inhibits the TGF-β/Smad pathway or reduces Ang II content, diminishes AT1R and Ang II binding, and inhibits the Ang II-AT1R-TGF-β pathway. These actions contribute to the mitigation of renal fibrosis development in hypertensive conditions. In aging kidneys, exercise improves renal fibrosis by inhibiting the TGF-β1/TAK1/MKK3/p38 MAPK signaling pathway, enhancing autophagy activity, and delaying the epithelial–mesenchymal transition, or activating PPAR α to reduce oxidative stress, inflammation, and lipid accumulation by inhibiting the expression of miR-21 and miR-34a. TGF-β1: transforming growth factor-β1; TAK1: transforming growth factor-β (TGF-β)-activated kinase 1; MKK3: mitogen-activated protein kinase (MAPK) kinase; p38MAPK: p38 mitogen-activated protein kinase; NOX4: NADPH oxidase 4; ROS: reactive oxygen species; NF-κB: nuclear factor kappa-B; NLPR3: NOD-like receptor thermal protein domain associated protein 3; AngⅡ: angiotensin II; AT1R: Ang II-angiotensin II type I receptor; α-SMA: α-smooth muscle actin; CTGF: connective tissue growth factor; Sirt1: silent information regulator 1; H_2_S: hydrogen sulfide; PGC-1α: peroxisome proliferator-activated receptor gamma coactivator 1-α; miR-21: microRNA-21; miR-34a: microRNA-34a. Created with Figdraw (www.figdraw.com), license ID: TRPRT70cb4.

In summary, moderate-intensity aerobic exercise demonstrates superior effectiveness in ameliorating hypertension-induced renal fibrosis, diabetic renal fibrosis, and aging-induced renal fibrosis. It achieves this by mitigating inflammation and oxidative stress, enhancing autophagy activity, and inhibiting the TGF-β pathway. However, high-intensity exercise exacerbates renal fibrosis, while the impact of low-intensity exercise on renal fibrosis remains unclear and warrants further exploration (Figure 3).

### 3.3. Myocardial Fibrosis

#### 3.3.1. Effect of Exercise on Myocardial Fibrosis

Myocardial fibrosis primarily arises from an imbalance between the accumulation of myocardial fibroblasts and the production and degradation of extracellular matrix, resulting in excessive ECM deposition and scarring. This process further impairs the normal systolic and diastolic function of the heart [90]. It is noteworthy that myocardial fibrosis is a common pathological feature observed in various cardiovascular diseases, including hypertension, myocardial infarction, diabetes mellitus, and heart failure [91]. The treatment of myocardial fibrosis holds paramount importance in enhancing the quality of life of patients with cardiovascular disease. Exercise interventions play a crucial role in slowing the progression of myocardial fibrosis, preventing myocardial damage, and improving cardiac function. Studies by Soori et al. have demonstrated that both 6 weeks of high-intensity interval training (HIIT) and continuous exercise training attenuated age-related myocardial fibrosis [92], even when exercise was initiated in late middle age [93]. Moreover, aerobic exercise exhibits a remarkable inhibitory effect on myocardial fibrosis, associated with diabetic cardiomyopathy and arthritis [94,95,96]. A recent study demonstrated that exercise can effectively stimulate endothelial progenitor cells to secrete and release exosomes, upregulate miR-126 expression, and further target TGF-β, which may serve as a potential modality to ameliorate myocardial fibrosis by decreasing cardiomyocyte apoptosis and inhibiting cardiac fibroblast transdifferentiation [97]. These findings collectively underscore the significant potential of exercise as an effective intervention strategy for mitigating myocardial fibrosis.

#### 3.3.2. Mechanisms of Exercise Regulation of Myocardial Fibrosis

Aging is accompanied by progressive and adverse cardiac remodeling, characterized by myocardial hypertrophy, fibrosis, and dysfunction. Experimental studies in animals have revealed an increment of collagen deposition in the left ventricle of aging rats, accompanied by a reduction in MMP-2 activity and elevated levels of COL-I, TGF-β1, and TIMP-1 mRNA expression. Interestingly, high-intensity resistance exercise (ladder climbing with 65, 85, 95, and 100% load, three times/week for 12 weeks) has been found to mitigate the accumulation of left ventricular collagen in aging rats, while simultaneously increasing MMP-2 activity and reducing the expression of COL-I, TGF-β1, and TIMP-1 [98]. This finding suggests that resistance exercise can protect cardiac structure and function by ameliorating left ventricular fibrosis in aged rats. Moreover, fibroblast growth factor 2 (FGF-2) induces the proliferation of cardiac fibroblasts through the activation of urokinase-type plasminogen activator (uPA) and MMP-2 expression, thereby contributing to cardiac fibrosis. Twelve weeks of swimming exercise (60 min/day, five times/week for 12 weeks) has been shown to improve cardiac fibrosis in aging rats by inhibiting the FGF2/uPA/MMP2 signaling pathway [30]. Recently, hydrogen sulfide (H2S) has been identified as a key factor that can affect the intracellular processes related to aging and is considered a potential target for preventing cardiovascular diseases. Aerobic exercise (60 min/day, 12 weeks) has been found to delay the development of cardiac fibrosis in senescent rats by restoring endogenous H_2_S levels in the senescent cardiac tissue [64]. Thus, exercise may attenuate senescent cardiac fibrosis by modulating various factors and signaling pathways.

Acute myocardial infarction (MI) triggered by coronary artery occlusion often leads to myocardial fibrosis. Exercise training (50 min/day, three or five times/week for 8 weeks) has been reported to alleviate cardiac fibrosis and ameliorate heart dysfunction after MI. For instance, eight weeks of aerobic exercise and aerobic resistance training effectively mitigated cardiac fibrosis in rats or mice post-MI by inhibiting the TGF-β1/Smad signaling pathway [99,100]. Research has reported that multiple miRNAs play important roles in myocardial fibrosis; intermittent aerobic exercise (60 min/day, five times/week for 8 weeks) can inhibit the TGF-β pathway by upregulating the expression of miR-29a and miR-101a, and ultimately reduce fibrosis and scar formation in the cardiac tissue of MI rats [101]. Fibroblast growth factor 21 (FGF21) exerts a protective effect on the infarcted heart. Ma et al. demonstrated that both aerobic (60 min/day, 4 weeks) and resistance exercises (75% of the maximum load, 4 weeks) could effectively suppress the TGF-β1-Smad2/3-MMP2/9 signaling pathway by enhancing the expression of FGF21, and then weaken myocardial fibrosis, oxidative stress, and apoptosis in myocardial infarction mice, ultimately improving cardiac function [102]. Meanwhile, the knockout of FGF21 weakened the cardioprotective effects of aerobic exercises after MI. This suggests that FGF21 plays an important role in the anti-fibrosis effect of exercise training [102]. Moreover, 4 weeks of moderate intensity aerobic exercise activates the Neuregulin 1 (NRG-1)/ErbB Signaling Pathway, promoting cardiomyocyte (CM) proliferation while inhibiting CM apoptosis. This exercise-induced modulation reduced collagen deposition and improved myocardial fibrosis in rats following myocardial infarction [103]. Abnormal mitochondrial quality control was found to be significantly associated with cardiac fibrosis [104]. Four weeks of aerobic exercise (60 min/day, 4 weeks) improved mitochondrial biogenesis and myocardial fibrosis by activating the Sirt1/PGC-1 α/PI3K/Akt signaling pathway and alleviated myocardial fibrosis in infarcted mice [105]. Iysocardiolipin acyltransferase-1 (ALCAT1) is a key enzyme that regulates cardiolipin metabolism and mitochondrial function; silencing ALCAT1 expression suppresses ROS overproduction and attenuates mitochondrial dysfunction [106]. Four weeks of aerobic exercise (40 min/day, five times/week for 4 weeks) decreased ALCAT1 expression and oxidative stress levels in the myocardium and serum, and subsequently inhibited cardiomyocyte apoptosis to improve cardiac function and myocardial fibrosis in MI rats [107]. Furthermore, a short-duration swimming exercise (15 min/day, five times/week for 8 weeks) may regulate mitochondrial quality control and reduce apoptosis by enhancing Sirt3 protein levels and improve myocardial fibrosis in MI mice [108]. Additionally, exercise can protect against myocardial fibrosis induced by myocardial infarction by inducing the expression of muscle cytokines. One study indicated that four weeks of resistance exercise (8 sets/day, five times/week for 4 weeks) suppresses myocardial fibrosis following myocardial infarction via the upregulation of irisin expression, the activation of AMPK-Sirt1, and the inactivation of TGFβ1-Smad2/3 [109]. Aerobic exercise (60 min/day, five times/week for 4 weeks) can also improve myocardial fibrosis in MI rats by promoting the skeletal muscle secretion of chitinase-3-like protein 1 (CHI3L1) and follistatin-like1 (FSTL1) [110,111]. These studies suggest that exercise may be an optimal method to improve myocardial fibrosis induced by myocardial infarction.

Metabolic disorders like obesity and diabetes are known contributors to myocardial fibrosis, a key pathological feature of end-stage diabetic cardiomyopathy (DCM) [112] that is closely associated with inflammation, oxidative stress, mitochondrial dysfunction, and apoptosis [113]. Wang et al. showed that 8 weeks of moderate-intensity aerobic exercise (60 min/day, five times/week for 8 weeks) effectively reduced myocardial fibrosis in diabetic rats by inhibiting the TGF-β1/Smad signaling pathway [114]. The hyperglycemia-induced production of ROS activated TGF-β1, thereby mediating fibrosis in diabetic cardiomyopathy [115]. Meanwhile, long-term moderate-intensity exercise countered this effect by reducing cardiac ROS production and oxidative stress levels, which in turn inhibited the expression of pro-fibrotic factors such as TGF-β1, MMP-2/9, CTGF, TIMP-1, as well as collagen types I and III, ultimately leading to an improvement in myocardial fibrosis [114]. In addition, 12 weeks of aerobic exercise (five times/week for 12 weeks) can reduce myocardial fibrosis by enhancing the expression of miR-486a-5p [116] or by inhibiting the protein expression of P2X7R [117] in cardiomyocytes, thereby inhibiting cardiomyocyte apoptosis. However, relatively few studies have focused on the effects of altered mitochondrial function. Wang et al. demonstrated that 15 weeks of aerobic exercise (60 min/day, 15 weeks) upregulates PGC-1 α and Akt expression in the heart of db/db mice, improving mitochondrial biogenesis and reducing cardiomyocyte apoptosis, thereby alleviating myocardial fibrosis [95]. Moreover, obesity-induced inflammation and oxidative stress can exacerbate cardiac fibrosis. Exercise ameliorated myocardial fibrosis in obese mice by inhibiting myocardial oxidative stress and promoting the expression of heme oxygenase 1 (HO-1) [118]. The latest study on the topic found that irisin (exerkines) also plays a protective role in diabetic myocardial fibrosis. Eight weeks of swimming exercise (60 min/day, five times/week for 8 weeks) activated the Sirt1/PGC-1 α/FNDC 5 signaling pathway through the inhibition of miR-34a expression, which promoted irisin secretion, reduced the expression of fibrosis markers (COL-1, COL-3, and TGF- β 1), and improved myocardial fibrosis in type 2 diabetic rats [119]. These findings underscore the therapeutic potential of exercise in mitigating myocardial fibrosis associated with metabolic disorders like obesity and diabetes, offering a promising avenue for managing diabetic cardiomyopathy and related complications.

In hypertensive conditions, increased cardiac load contributes to myocardial fibrosis. Studies have reported that exercise can effectively improve hypertensive-induced myocardial fibrosis through various mechanisms. One study reported that exercise promoted the expression of CCDC80tide, inhibited Janus kinase 2 (JAK) activity, and activated signal transducer and activator of transcription 3 (STAT3), ultimately leading to an improvement in myocardial fibrosis [120]. Additionally, Hong et al. found that aerobic exercise (60 min/day, five times/week for 12 weeks) ameliorated myocardial fibrosis in aged hypertensive rats by inhibiting the lysyl oxidase-like 2 (LOXL-2)/TGF-β signaling pathway, as well as the expression of AT1R and FGF23 [121]. Furthermore, swimming exercise (60 min/day, 8 weeks) was shown to protect the myocardium from hypertensive injury by alleviating myocardial fibrosis in hypertensive rats through the activation of the AMP-activated protein kinase α1 (AMPKα1)/Sirt1/peroxisome proliferator-activated receptor gamma coactivator 1-α (PGC-1α) pathway [122].

Furthermore, myocardial fibrosis is closely linked to inflammation and oxidative stress, particularly due to estrogen deficiency in postmenopausal females [123,124]. Regular aerobic exercise (60 min/day, five times/week for 13 weeks; 60 min/day, 10 weeks) has been shown to mitigate myocardial fibrosis induced by ovariectomy in animal models [125,126]. Lin et al. demonstrated that eight weeks of aerobic exercise (60 min/day, five days/week) significantly reduced ovariectomy-induced myocardial fibrosis in hypertensive rats. This improvement was achieved by downregulating AT1R expression, inhibiting the TNF-α/NF-κB and TGF-β/CTGF signaling pathways, and decreasing the levels of MMP-9 and COL1 [127]. In addition, cardiomyocyte apoptosis is a critical pathological mechanism in the progression of heart failure. Aerobic exercise for 10 weeks (60 min/day, five days/week) was found to attenuate ovariectomy-induced myocardial fibrosis in rats by exerting anti-apoptotic effects. These effects were mediated through the inhibition of Fas receptor-dependent and mitochondria-dependent apoptosis pathways [128]. MicroRNAs (miRNAs) are emerging as potential therapeutic targets for cardiovascular diseases. Moderate-intensity swimming exercise (60 min/day, six days/week for eight weeks) has been shown to ameliorate cardiac fibrosis in ovariectomized type 2 diabetic rats. This effect was associated with increased levels of miR-133 and Bcl-2, along with a reduced expression of pro-apoptotic proteins such as Bax and caspases in myocardial tissue [129]. Furthermore, a similar exercise regimen (60 min/day, six days/week for eight weeks) improved myocardial fibrosis in ovariectomized rats by enhancing the expression of miR-29 and IGF-1 in cardiac tissue [130].

**Figure 4:**
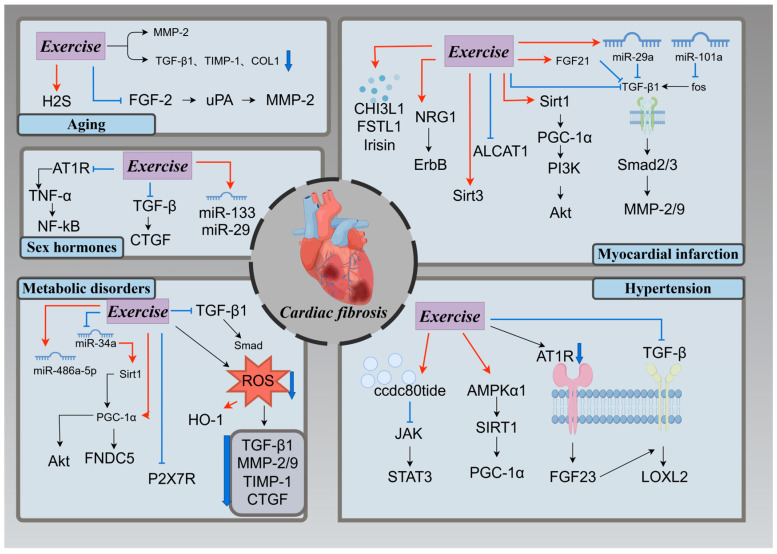
Mechanisms of exercise modulation of myocardial fibrosis. Aging, myocardial infarction, metabolic diseases, and hypertension can all lead to myocardial fibrosis. Aging: Exercise reduces collagen deposition by enhancing MMP-2 activity and decreasing the expression of fibrosis-associated factors (TGF-β1, TIMP-1, COL-I). It also restores endogenous H_2_S levels or inhibits the FGF-2/uPA/MMP-2 signaling pathway. Metabolic diseases: Exercise inhibits the TGF-β1/Smad signaling pathway or reduces ROS production, promoting HO-1 expression and inhibiting fibrosis-related factors. Myocardial infarction: Exercise inhibits TGF-β1 signaling through the NRG-1/ErbB signaling pathway or by upregulating miR-29a, miR-101a, and FGF-21 expression. In addition, exercise can also secrete several myokines such as irisin, CHI3L1, and FSTL1 to ameliorate myocardial fibrosis after myocardial infarction. Hypertension: Exercise attenuates myocardial fibrosis by inhibiting the LOXL-2/TGF-β signaling pathway and the expression of AT1R and FGF23, or by promoting the expression of ccdc80tide and AMPKα1. MMP: matrix metallopeptidase; TGF-β1: transforming growth factor-β1; TIMP-1: tissue inhibitor of metal protease1; H2S: hydrogen sulfide; COL-Ⅰ: Collagen 1; FGF-2: fibroblast growth factor 2; uPA: urokinase-type plasminogen activator; NRG1: Neuregulin 1; FGF-21: fibroblast Growth Factor 21; miR-29a: microRNA-29a; miR-101a: microRNA-101a; smad: small mother decapentaplegic protein; ROS: reactive oxygen species; CTGF: connective tissue growth factor; HO-1: heme oxygenase 1; JAK: janus kinase 2; STAT3: signal transducer and activator of transcription 3; AMPKα1: AMP-activated protein kinase α1; Sirt1: silent information regulator 1; PGC-1α: peroxisome proliferator-activated receptor gamma coactivator 1-α; AT1R: Ang II-angiotensin II type I receptor; FGF-23: fibroblast Growth Factor 23; LOXL2: Lysyl oxidase-like 2; miR-34a: microRNA-34a; miR-486a-5p: microRNA-486a-5p; miR-29: microRNA-29; miR-133: microRNA-133; TNF-α: tumor necrosis factor; NF-kB: nuclear factor kappa-B; AKT: protein kinase B; PI3K: phosphoinositide 3-kinase; ALCAT1: lysocardiolipin acyltransferase-1. Created with Figdraw (www.figdraw.com), license ID: PRPRW1c841.

In summary, exercise attenuates myocardial fibrosis by enhancing myocardial antioxidant capacity, reducing reactive oxygen species production, diminishing inflammatory responses, and inhibiting the TGF-β signaling pathway (Figure 4). These findings highlight the multifaceted benefits of exercise in combating myocardial fibrosis.

### 3.4. Skeletal Muscle Fibrosis

#### 3.4.1. Effects of Exercise on Skeletal Muscle Fibrosis

Skeletal muscle fibrosis, characterized by excessive collagen deposition, is common in conditions like muscular dystrophies [131], aging [132], and severe muscle damage [133]. Exercise has shown effectiveness in alleviating skeletal muscle fibrosis. For instance, 4 weeks of aerobic exercise significantly reduces fibrotic areas in the muscles of ZSF1 rats [134]. In conditions such as aging and obesity, exercise helps prevent excessive collagen deposition by decreasing collagen synthesis and down-regulating MMP activity, thereby slowing down the progression of skeletal muscle fibrosis [135,136]. Exercise emerges as a valuable intervention for mitigating skeletal muscle fibrosis, offering promising prospects for managing conditions associated with this pathological feature.

#### 3.4.2. Mechanisms of Exercise Regulation of Skeletal Muscle Fibrosis

Aging, chronic diseases, and poor lifestyles often lead to primary or secondary sarcopenia, accompanied by skeletal muscles fibrosis [137]. C1q, a component of the complement system, increases with age. The C1q-C1r-C1s complex binds to Frizzled, activating the Wnt/β-catenin pathway and promoting fibrosis development [138]. It was shown that 12 weeks of resistance training (three times/week, 12 weeks) reduced the fibrotic area in the tibialis anterior muscle of aged mice by decreasing C1q expression and blocking Wnt/β-catenin pathway activation [19]. This suggests that resistance exercise mitigates aging-induced skeletal muscle fibrosis by inhibiting the Wnt/β-catenin signaling pathway, offering potential therapeutic benefits for sarcopenia-related conditions.

Diabetic patients often exhibit impaired glucose uptake and metabolism in skeletal muscle, leading to increased collagen deposition and fibrosis [139]. A study by Amani et al. demonstrated that 6 weeks of aerobic exercise (30 min/day, five times/week for 6 weeks) in STZ-induced diabetic rats reduced blood glucose levels and decreased the protein content of NRG1 and ErbB2. This intervention significantly reduced the fibrotic area in the muscle, such as the extensor digitorum longus and soleus [23]. Exercise also mitigates muscle fibrosis induced by high-fat diets. Twelve weeks of aerobic exercise (40 min/day, five times/week for 12 weeks) attenuated fibrosis in mouse gastrocnemius muscle induced by high-fat diets by inhibiting the expression levels of TGF-β1, Smad3, and COL-I in muscle tissue [136]. These findings suggest that exercise is effective in ameliorating skeletal muscle fibrosis in diabetes and high-fat-diet-induced models by improving glucose metabolism, reducing fibrotic signaling pathways, and lowering collagen expression levels in muscle tissue.

Duchenne muscular dystrophy (DMD) is characterized by recurrent muscle fiber damage, chronic inflammation, and progressive fibrosis. mdx mice, commonly used as a model for DMD, exhibit similar pathological features [140,141]. It was found that low-intensity exercise (9 m/min, 30 min/d, 3 times/week) significantly reduced the area of collagen deposition in the gastrocnemius and tibialis anterior muscles of mdx mice [142,143]. This suggests that low-intensity exercise can effectively mitigate skeletal muscle fibrosis in mdx mice. Conversely, high-intensity exercise has been associated with adverse effects on skeletal muscle fibrosis in mdx mice. High-intensity exercise leads to significant increases in the expression levels of fibrotic markers such as TGF-β1, Smad2/3, CTGF, COL-I, and COL-III in skeletal muscle tissue, which exacerbates collagen deposition and worsens skeletal muscle fibrosis in mdx mice [144,145,146,147]. In addition, swimming and treadmill exercise with an upward inclination of 7° have also been found to adversely affect skeletal muscle fibrosis in mdx mice [148,149,150]. These findings collectively suggest that moderate-to-high-intensity exercise exacerbates skeletal muscle fibrosis in mdx mice, while low-intensity exercise shows promise in ameliorating fibrosis in this model.

In summary, exercise exhibits promising effects in ameliorating skeletal muscle fibrosis in various conditions, such as aging, diabetes, and high-fat-induced fibrosis. These beneficial effects are often mediated through the targeting of key signaling pathways, such as the TGF-β1 or Wnt pathways. However, when considering skeletal muscle fibrosis in mdx mice, the impact of exercise appears to vary depending on the intensity of the exercise regimen. Low-intensity exercise shows promise in reducing fibrosis in mdx mice, whereas moderate-to-high-intensity exercise exacerbates skeletal muscle fibrosis in this model. These findings underscore the importance of tailoring exercise interventions based on the specific condition and individual needs, especially in the context of muscular dystrophies like DMD.

### 3.5. Liver Fibrosis

#### 3.5.1. Effects of Exercise on Liver Fibrosis

Liver fibrosis is a hallmark of various chronic liver diseases, including viral hepatitis, alcoholic fatty liver disease, non-alcoholic fatty liver disease (NAFLD), and cholestasis. As these conditions progress, hepatic tissue damage and repair become dysregulated, leading to the activation of hepatic stellate cells (HSCs) and the excessive accumulation of extracellular matrix, ultimately resulting in liver fibrosis and cirrhosis [151,152]. Studies have demonstrated that 12 weeks of moderate-to-vigorous aerobic exercise significantly improves liver fibrosis in patients with NAFLD [153]. Additionally, exercise has been shown to ameliorate hepatic fibrosis in individuals with metabolic syndrome [154], highlighting the potential of exercise as an effective intervention for improving liver fibrosis across various liver diseases.

#### 3.5.2. Mechanisms of Exercise Regulation of Liver Fibrosis

Exercise exerts its beneficial effects on liver fibrosis through various mechanisms. One such mechanism involves the modulation of hepatic macrophage infiltration. One study has shown that blocking macrophage infiltration effectively inhibits the activation of hepatic stellate cells and ameliorates hepatic fibrosis [155]. Another study indicated that 16-week aerobic exercise reduced hepatic TNF-α mRNA levels, attenuated inflammation, and suppressed hepatic fibrosis markers such as TGF-β1, TIMP-1, COL-Ⅰ, and α-SMA mRNA expression. Additionally, exercise reduced collagen deposition and decreased the expression of macrophage markers (F4/80-positive cells) and chemokines (MCP-1, CXCL14) in the liver [15]. However, the effectiveness of exercise in alleviating liver fibrosis may vary depending on the intensity of exercise and the underlying cause of liver disease. For instance, moderate-intensity exercise did not significantly alleviate Western diet-induced liver fibrosis in rats [156], while HIIT showed superior effects in reducing liver inflammation and fibrosis in NASH mice compared to moderate-intensity exercise [157]. Further research is needed to elucidate the precise mechanisms underlying the effects of exercise on liver fibrosis and to explore its potential therapeutic benefits in other liver diseases.

## 4. Conclusions and Perspectives

**Figure 5:**
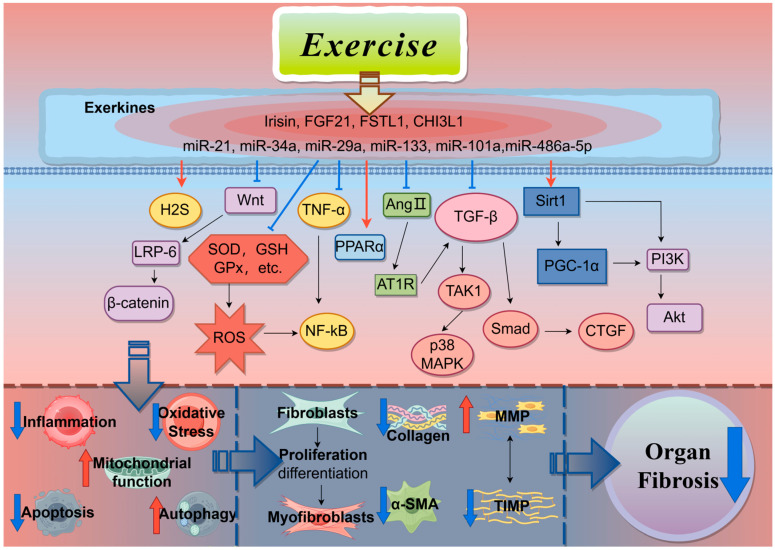
Antifibrotic mechanisms of exercise: Exercise exerts its anti-fibrotic effects by directly or indirectly (secreting exerkines or targeting microRNAs) affecting multiple signaling pathways associated with fibrogenesis. Created with Figdraw (www.figdraw.com), license ID: IYAUA656e8.

This paper provides a comprehensive overview of the recent advancements in understanding the role of exercise in the prevention and treatment of organ fibrosis. Accumulating evidence suggests that exercise has beneficial effects on fibrotic diseases affecting various organs, including the heart, liver, lung, kidney, and skeletal muscle. These effects are mediated through the modulation of myofibroblast activation, autophagy, oxidative stress, and inflammatory responses, primarily via signaling pathways such as TGF-β1/Smad, Wnt/β-catenin, ROS, NF-κB, sirtuins, and miRNAs (miR-126, miR-29a, miR-101a, miR-34a, miR-21, miR-133, miR-486a-5p). However, studies examining the antifibrotic properties of exercise are mainly based on animal studies and cell models, and clinical experimental evidence is lacking. One of the most difficult obstacles is assigning a personalized exercise regimen for the patient. Therefore, the type of exercise and the duration of exercise still need further research to involve reasonable clinical trials and expand the scope of application. Meanwhile, the current understanding of the molecular mechanisms underlying exercise-induced anti-fibrotic effects remains insufficient. Exerkines, bioactive molecules secreted by tissues/organs during exercise, including proteins/polypeptides, RNAs, and metabolic products, play crucial roles in mediating the benefits of exercise. While exercise has been shown to ameliorate cardiac fibrosis induced by myocardial infarction by regulating FGF21, irisin, FSTL1, and CHI3L1, it is yet to be elucidated whether exercise plays a significant role in improving fibrosis in other organs by regulating these factors (Figure 5). Furthermore, it is currently unclear whether other exerkines mediate the anti-fibrotic effect of exercise. The interaction between exerkines and the crosstalk between organs are currently research hotspots. Whether exercise exerts its anti-fibrosis effect through the interaction between exerkines or crosstalk between organs remains to be further investigated. Moreover, exerkines are also regulated by the same transcription factor, and key transcription factors may be important targets for future studies on the mechanisms by which exercise improves organ fibrosis.
